# Moxibustion Modulates ALOX15‐Mediated Lipid Peroxidation to Inhibit Ferroptosis in Synovial Inflammatory Injury of Rheumatoid Arthritis

**DOI:** 10.1155/mi/4516938

**Published:** 2026-07-02

**Authors:** Tiancheng Wang, Chuanyue Peng, Qiannan Liu, Zanchen Zhou, Dong Gao, Yifan Li, Meilin Zhang, Feng Hao, Chuanyu Peng

**Affiliations:** ^1^ Anhui University of Chinese Medicine Anhui Province Key Laboratory of Meridian Viscera Correlation Ship Hefei Anhui China; ^2^ Hong Kong Baptist University School of Chinese Medicine Kowloon Tong Kowloon Hong Kong; ^3^ Anhui Medical University Clinic Medical College Hefei Anhui China; ^4^ Anhui University of Chinese Medicine School of Acupuncture-Moxibustion and Tuina Hefei Anhui China; ^5^ The First Affiliated Hospital of Anhui University of Chinese Medicine Hefei Anhui China ahtcm.edu.cn; ^6^ Nanjing University of Chinese Medicine School of Acupuncture-Moxibustion and Tuina and School of Health Preservation and Rehabilitation Nanjing Jiangsu China

**Keywords:** ALOX15, arachidonic acid, ferroptosis, lipid peroxidation, moxibustion, rheumatoid arthritis

## Abstract

Rheumatoid arthritis (RA) is a chronic inflammatory autoimmune disease characterized by synovial, cartilage, and bone damage. Emerging research evidence has linked its pathogenesis to ferroptosis, arachidonic acid (ARA) metabolism, and lipid peroxidation. Lipid peroxidation serves as the final executor of ferroptosis, and arachidonate lipoxygenase (ALOX)–mediated oxidative reactions enzymatically promote lipid peroxidation. Moxibustion (MOX), a traditional therapeutic modality in Chinese medicine, has demonstrated significant efficacy in our study. Specifically, MOX applied at the Zusanli (ST36) and Shenshu (BL23) acupoints effectively ameliorated paw swelling in Freund’s complete adjuvant (FCA)–induced RA model rats, significantly reduced arthritis scores, and corrected ARA metabolic dysregulation. Furthermore, MOX treatment markedly decreased the expression levels of arachidonate 15‐lipoxygenase (ALOX15), acyl‐CoA synthetase long‐chain family member 4 (ACSL4), lysophosphatidylcholine acyltransferase 3 (LPCAT3), and reactive oxygen species (ROS) in the synovial tissues of RA model rats while increasing the expression of ferritin heavy chain 1 (FTH1) and glutathione peroxidase 4 (GPX4). Serum analyses revealed significant reductions in malondialdehyde (MDA), lipid peroxide (LPO), interleukin‐12 (IL‐12), and tumor necrosis factor‐alpha (TNF‐α) levels, alongside elevated glutathione (GSH) and superoxide dismutase (SOD) levels. The underlying mechanism involves the modulation of ALOX15‐mediated lipid peroxidation to inhibit ferroptosis, thereby alleviating RA‐associated inflammatory damage. These findings highlight the substantial therapeutic potential of MOX in mitigating RA‐related inflammation and provide a novel theoretical basis for its clinical application in RA management.

## 1. Introduction

Rheumatoid arthritis (RA), an autoimmune disease, primarily manifests as aggressive and symmetrical polyarthritis [1]. The synovial tissue within the affected joint cavity can generate an invasive pannus, leading to recurrent synovial inflammation, joint cartilage destruction, joint deformity, and functional loss. This condition severely impairs the quality of life of patients and imposes a massive burden on their families and society [2]. Because of its complex etiology and pathogenesis as well as the difficulty in achieving a lifelong cure, RA as a refractory disease has received widespread attention in the medical community.

Ferroptosis, an iron‐mediated programmed cell death mode, is primarily induced by reactive oxygen species (ROS) accumulation. It manifests as widespread lipid peroxidation and severe mitochondrial and cell membrane dysfunction [3]. Studies conducted by Doll et al. [4] and other researchers have shown that a broad spectrum of polyunsaturated fatty acids (PUFAs), for example, arachidonic acid (ARA), may be involved in the ferroptosis pathway. ARA metabolism primarily occurs through three pathways: cycloxygenase (COX), lipoxygenase (LOX), and cytochrome P450 (CYP450) [5]. Lipid peroxidation is the ultimate inducer of ferroptosis, and the oxidation reaction mediated by arachidonate LOX (ALOX) promotes lipid peroxidation through an enzymatic reaction. Inflammation is a fundamental pathological process in which the body responds to various damaging factors, primarily for defense purposes [6]. Ferroptosis is closely associated with inflammatory responses, as the ferroptosis process generates certain inflammatory factors related to peroxide metabolism and ARA metabolism. To date, several studies have confirmed the crucial role of ferroptosis in RA. However, there is a scarcity of data regarding the effect of ALOX‐induced lipid peroxidation and the ferroptosis pathway in Freund’s complete adjuvant (FCA)–induced RA.

Currently, Western medicine treats RA primarily through corticosteroids, nonsteroidal anti‐inflammatory drugs, and antirheumatic drugs, which frequently exert substantial side effects and can easily lead to drug resistance [7]. Therefore, there is an urgent requirement to find novel approaches to alleviate articular cartilage destruction, reduce inflammatory damage, and enhance the quality of life and subjective health of patients with RA. The main mechanism underlying the effect of traditional Chinese medicine (TCM) on RA includes warming the meridians, dispersing cold, dredging the meridians, and stopping pain; this is consistent with the function of moxibustion (MOX), a characteristic therapy of TCM. MOX therapy involves the direct or indirect burning and warming of acupuncture points on the body surface by using moxa sticks (fresh moxa leaves were dried, crushed, and sieved; and other processes were used to make moxa velvet, which is then rolled into cotton paper to form cylindrical moxa sticks). Through the transmission of meridians, the gentle heat of fire and the effect of medicinal herbs work synergistically to warm and invigorate qi and blood, support health, and eliminate pathogens, thus achieving therapeutic and healthcare effects [8]. Numerous clinical reports and experimental animal studies have reported that MOX therapy is remarkably effective for RA treatment [9, 10]. Clinically, MOX application in treating RA can alleviate the symptoms, control the disease, and restore joint function. In experimental animals, MOX has a distinct anti‐inflammatory and immunomodulatory effect on RA model rats, wherein it inhibits the expression of inflammatory cytokines, upregulates the expression of anti‐inflammatory factors, and induces an anti‐inflammatory effect through signaling pathways such as Janus kinase‐signal transducer and activator of transcription, nuclear factor kappa‐B, and Ras‐mitogen–activated protein kinase. MOX application to prevent and treat RA demonstrates increasing advantages by not only providing comprehensive regulation of the body but also offering the unique benefits of nontoxicity and no side effects.

In preliminary studies, our research group found that ferroptosis‐related factors such as p53, glutathione peroxidase 4 (GPX4), and solute carrier family 7 member 11 are involved in the inflammatory response of RA model rats [11]. MOX can suppress ferroptosis occurrence by controlling the expression of ferroptosis‐related factors, thereby improving inflammatory damage in the synovial tissue of RA model rats. However, it remains unclear whether MOX’s therapeutic effect on RA and its underlying mechanism are related to lipid peroxidation inhibition and ferroptosis regulation.

This study aimed to test whether MOX alleviates RA‐associated synovial inflammatory injury by modulating arachidonate 15‐LOX (ALOX15)–mediated lipid peroxidation and thereby inhibiting ferroptosis; to this end, we employed methods such as targeted metabolomics, hematoxylin and eosin (HE) staining, immunohistochemistry (IHC), and western blot (WB) analyses in FCA‐induced RA rats, in order to further elucidate the partial mechanism of action of MOX in treating RA and provide important theoretical basis and practical significance.

## 2. Materials and Methods

### 2.1. Details of Animal Model Development and Intervention Methods

**Figure 1:**
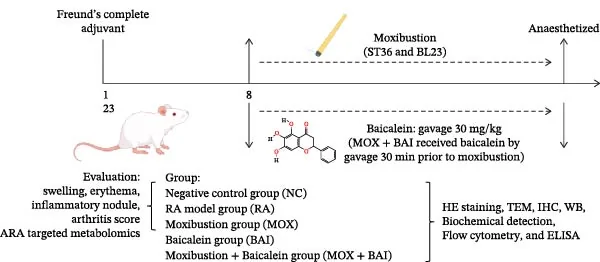
Study schedule and design.

Thirty male clean SD rats (weight: 200 ± 20 g) were provided by Liaoning Changsheng Biotech Co., Ltd. (production license number: SCXK [Liaoning 2020‐0001]). The rats were maintained in the animal laboratory of Anhui University of Chinese Medicine under the following conditions: constant temperature (22 ± 2°C), 12 h light/dark cycle, relative humidity: 65% ± 5%, and free access to a standard diet and water. Following 1 week of adaptive feeding, the rats were assigned randomly to the following groups with sx rats per group: normal control (NC), RA, MOX, baicalein (BAI), and MOX + BAI (MOX + BAI). On the first day of the experiment, 0.5 mg/kg of FCA injection (Sigma, USA) was administered into the right hind paw to induce inflammation and establish the RA model [12]. Intervention started on the 7th day after model establishment. Based on the animal acupoint chart for experimental acupuncture and MOX, Zusanli (ST36) and Shenshu (BL23) acupoints were selected. In rats, ST36 was located ~5 mm distal to the fibular head and 2–3 mm lateral to the anterior crest of the tibia at the belly of the tibialis anterior; BL23 was located at the level of the lower border of the second lumbar spinous process, 5–7 mm lateral to the posterior midline over the erector spinae. In the MOX group, moxa sticks were hung on the MOX net of the MOX stand for 20 min at a distance of 2 cm from the acupoints during each treatment session (on both sides), and both acupoints were stimulated alternately once a day. The BAI (Shanghai Yuanye Biotechnology Co., Ltd., B20571) group received 30 mg/kg of the ALOX15 inhibitor BAI through gavage [13]. The MOX + BAI group received BAI 30 min before MOX and then underwent the same intervention as the MOX group [14]. NC and RA group rats were kept on a specialized wooden frame for 20 min. Each group received intervention once a day for 15 days [15]. A complete study schedule and timeline are provided in Figure 1. During the experiment, all animals were managed by strictly following the relevant regulations of the “Guiding Opinions on the Kind Treatment of Experimental Animals.” The Animal Ethics Committee of Anhui University of Chinese Medicine approved this study (Approval Number AHUCM‐rats‐2024013). The swelling and redness of the footpads served as indirect indicators of severity due to inflammation and were used to evaluate the preparation process of RA models. Arthritis score was evaluated on a 0–4 scale: 0 point, no swelling or redness; 1 point, mild swelling or redness; 2 points, coexistence of moderate swelling and redness; 3 points, coexistence of severe swelling and redness; and 4 points, severe swelling and redness, accompanied by joint stiffness and substantially restricted mobility [16].

### 2.2. Sample Collection

After the intervention was completed, all rats were intraperitoneally anesthetized with 20% urethane solution (0.3 mL/100 g, Beijing Solarbio Science and Technology Co., Ltd., IE0570), which was within the recommended range for intraperitoneal anesthesia in rodents in the guidelines, and the depth of anesthesia was confirmed by the disappearance of the righting reflex and corneal reflex. Following confirmation of deep anesthesia, euthanasia was performed via exsanguination through blood collection from the abdominal aorta. This method is consistent with guidelines for humane euthanasia in rodents, as exsanguination under deep anesthesia induces rapid loss of consciousness and death without distress. After blood collection, the rats were confirmed to have no vital signs to ensure complete euthanasia. Subsequently, the collected blood was centrifuged in a refrigerated centrifuge (4°C, 3000 rpm) for 15 min with a centrifugal radius of 68 mm. Some portion of the serum was stored at −80°C. The right knee joints of rats from each group were longitudinally incised, and the skin and muscles were separated to expose the patella. The synovial tissue was then isolated and stored at −80°C.

### 2.3. Metabolite Extraction

Serum samples (100 µL) were collected from each rat in the NC, RA, and MOX groups and mixed with the BHT protein precipitant (500 µL) and 10 µL of 1 µg/mL internal standard. The mixture was vortexed for 20 s and subjected to centrifugation at 14,000 relative centrifugal force and 4°C for 10 min. Next, 400 µL of the collected supernatant was mixed with 1000 µL of pure water. Oasis HLB 96‐well plates were used for purifying the samples. The plates were activated with 2 mL of methanol (1 mL added each time), the volume was balanced with 2 mL of pure water (1 mL added each time), and the samples were loaded multiple times. Next, washing solution A (2 mL) was added twice (1 mL added each time), followed by twice addition of washing solution B (2 mL; 1 mL added each time). The filtrate was discarded. Next, the samples were eluted twice with pure methanol (1 mL). The eluent was collected, dried with nitrogen, and stored at −80°C.

### 2.4. Chromatographic Conditions

An ultrahigh‐performance liquid chromatography system (1290 Infinity III LC System, Agilent) was employed for chromatographic separation. The samples were kept in an autosampler maintained at 4°C, with a column temperature of 35°C. Mobile phase A and mobile phase B were 0.1% formic acid in water and 0.1% formic acid in acetonitrile, respectively. The injection volume and flow rate were 2 μL and 400 μL/min, respectively. The following liquid phase gradients were used: 0–1 min, phase B sustained at 30%; 1–9 min, phase B linearly varied from 30% to 90%; 9–11 min, phase B sustained at 90%; 11–11.1 min, phase B linearly varied from 90% to 20%; and 11.1–14 min, phase B sustained at 20%. Quality control (QC) samples were placed at regular intervals in the sample queue to monitor and assess whether the system was stable and reproducible. To correct the chromatographic retention time (RT), we also included a mixture of standard substances for the target analytes in the sample queue.

### 2.5. Mass Spectrometry

Mass spectrometry was conducted with a mass spectrometer (QTRAP 5500, SCIEX) in the negative ion mode. For generating negative ions, the following QTRAP 5500 ESI source conditions were applied: source temperature: 500°C; ion source gas 1:50; ion source gas 2:50; curtain gas: 30; and ion spray voltage floating: −4500 V. Analyte ion pairs were detected under the multiple reaction monitoring mode. The ion pair information for all target substances is presented in Table S1.

### 2.6. HE Staining

Synovial tissues from each group of rats were fixed with 4% paraformaldehyde solution, rinsed with pure water, dehydrated with ethanol, and embedded in paraffin after immersion in xylene. The tissues were then sectioned at 4 μm thickness, baked in a thermostat, deparaffinized, stained with hematoxylin, differentiated using 1% hydrochloric acid‐ethanol solution, stained with a bluing reagent and eosin dye, dehydrated, and mounted. Pathological and morphological changes in the synovial tissues of rats were detected by optical microscopy.

### 2.7. Transmission Electron Microscopy (TEM)

The synovial tissues were initially fixed with glutaraldehyde and then subjected to fixation with PBS and 1% osmium tetroxide at room temperature. Subsequently, the tissues were subjected to gradient ethanol dehydration, staining with uranium acetate in 70% ethanol, and epoxy resin infiltration and embedding, and 70‐nm‐thick sections were prepared. The sections were stained with lead citrate and uranium acetate and observed by TEM to examine ultrastructural changes in the mitochondrial morphology of synovial cells. TEM images were captured and collected.

### 2.8. IHC

Paraffin‐embedded tissue sections were subjected to deparaffinization and hydration. Subsequently, antigen retrieval was performed by treating the tissue sections with a citric acid antigen retrieval buffer, followed by blocking with serum. The sections were then incubated at 4°C overnight in a humidified chamber. Next, the sections were rinsed with PBS and incubated with secondary antibodies. DAB and hematoxylin were used for color development and counterstaining of the sections, respectively. The sections were then dehydrated and mounted. Images were captured and analyzed by microscopy. Semiquantitative analysis of IHC images was performed using ImagePro Plus 6.0 software, and the integrated absorbance (IA)/area was used as the detection result.

### 2.9. WB Assay

Briefly, 100 mg of synovial tissue was homogenized in 600 μL RIPA lysis buffer (Beyotime, P0013B) and subjected to centrifugation for 10 min at 12,000 rpm. The supernatant was collected and used for protein extraction. SDS‐PAGE gels were prepared, and samples were loaded, electrophoresed, and transferred to membranes. The membranes were subsequently blocked for 2 h with 5% nonfat milk powder at room temperature and further incubated at 4°C overnight with primary antibodies: ALOX15, 1:1000; acyl‐CoA synthetase long‐chain family member 4 (ACSL4), 1:5000; recombinant lysophosphatidylcholine acyltransferase 3 (LPCAT3), 1:2000; ferritin heavy chain 1 (FTH1), 1:1000; and GPX4, 1:2000 (Abcam). After incubation with secondary antibodies (1:20,000) and washing three times with PBST for 10 min each, the ECL ultrasensitive chemiluminescence reagent was added dropwise, and a gel imaging system was used for analyzing the net optical density and molecular weight of the target bands. The gray values of each protein band were quantitatively analyzed.

### 2.10. Biochemical Detection

Serum samples from each group of rats were collected and assayed using a malondialdehyde (MDA) detection kit, a lipid peroxide (LPO) assay kit, a superoxide dismutase (SOD) assay kit, and a glutathione (GSH) detection kit (all from Nanjing Jiancheng Bioengineering Institute; A003‐1, A160‐1, A001‐3, and A006‐2‐1, respectively) as recommended by the manufacturer. The absorbance values were measured at 532, 586, 450, and 405 nm wavelengths by using a microplate reader, and the contents were calculated.

### 2.11. Flow Cytometry

Spare synovial tissue samples from each group were cut into small pieces by using surgical scissors and washed twice with PBS by centrifugation for 6 min at 2500 rpm; the supernatant was then discarded. The tissue pieces were digested, and the process was repeated once. The precipitate was resuspended in PBS to yield a single‐cell suspension. Cells from each group were collected and adjusted to a cell density of 1^–10^ × 10^6^ cells/mL. A ROS detection kit (Beyotime, S0033S) was used, and DCFH‐DA was added at a 10 μmol/L concentration. The cells were incubated for 20 min at 37°C and stirred every 3–5 min to ensure adequate interaction between the probe and the cells. Nonstained cells were used as negative controls. The cells were washed two times with PBS to remove any uninternalized DCFH‐DA and analyzed for ROS by flow cytometry.

### 2.12. Enzyme‐Linked Immunosorbent Assay (ELISA)

Serum samples from each group of rats were assayed using interleukin‐12 (IL‐12) and tumor necrosis factor‐alpha (TNF‐α) ELISA kits (Wuhan Jiyinmei Technology Co., Ltd.; JYM1006Ra and JYM0635Ra, respectively) as recommended by the manufacturer. Standard dilution and sample addition, enzyme addition, incubation, solution preparation, washing, color development, and other operations were performed strictly following the kit instructions. A stop solution (50 μL) was added to terminate the reaction. A microplate reader was utilized for estimating the absorbance values of each well at 450 nm.

### 2.13. Statistical Analysis

Multiquant 3.0.2 software was employed for extracting chromatographic peak areas and RTs. To identify metabolites, we corrected RTs using the standards of the target substances. Statistical analyses were performed using GraphPad Prism 9.0. Data are presented as box‐and‐whisker plots showing the median and interquartile range (IQR) with all individual values overlaid for endpoints with *n* = 6 per group and as individual points with a median reference line for endpoints with *n* = 3 per group (IHC, WB, and ROS). Group comparisons were analyzed by two‐tailed *t*‐tests or one‐way ANOVA, as appropriate; when normality or homogeneity assumptions were not met, Kruskal–Wallis tests with Dunn’s post hoc were used. *p*‐Values < 0.05 were considered statistically significant.

## 3. Results

### 3.1. MOX Improves the Degree of Inflammation in RA Model Rats

**Figure 2:**
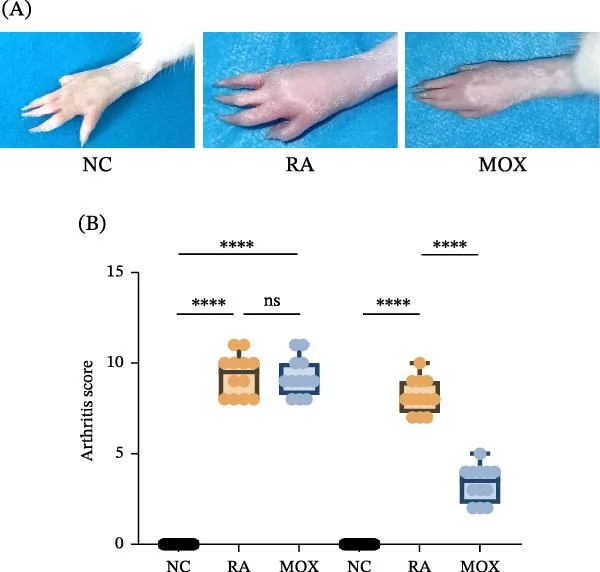
Moxibustion improves the degree of inflammation in RA model rats. (A) Morphological changes in the toe of rats in the NC, RA, and MOX groups. (B) Arthritis scores of NC, RA, and MOX group rats after modeling and therapy.  ^∗∗∗∗^ *p* < 0.0001.

The RA model was successfully established based on the emergence of acute inflammatory swelling in the toe, accompanied by secondary systemic polyarthritis or even erythema or inflammatory nodules in the forelimb or ear tail. Following model preparation, compared to NC group rats, RA and MOX group rats exhibited prominent redness and swelling in their feet, with a significant elevation in arthritis scores. At baseline (day 7 post‐FCA, before intervention), arthritis scores did not differ between the RA and MOX groups (*p* > 0.05), indicating comparable baseline severity; after 15 days of intervention, arthritis scores in MOX group rats were significantly lower than RA (*p* < 0.0001). These findings collectively demonstrate that MOX is efficacious in improving the degree of inflammation in RA model rats (Figure 2).

### 3.2. MOX Regulates ARA Metabolism in RA Model Rats

**Figure 3:**
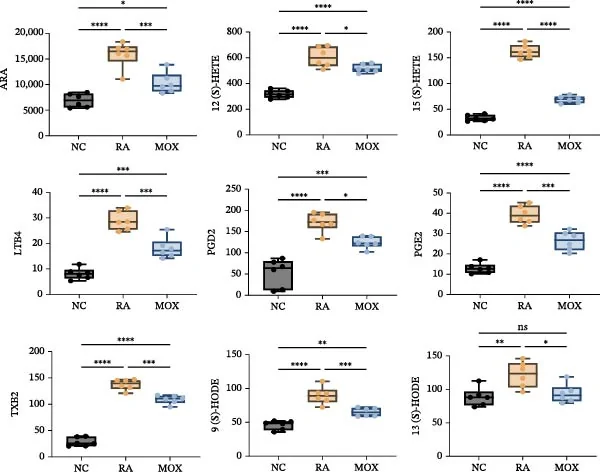
Serum levels of ARA and nine metabolites in the rats of the NC, RA, and MOX groups.

QC samples were obtained by mixing equal amounts of serum from the NC, RA, and MOX groups and utilized for evaluating whether the data were stable and reproducible. Table 1 shows the RT, coefficient of variation (CV), and concentration results of the analytes in the QC samples. A CV of <30% indicates that the data for the analytes in the QC samples are reliable and stable. Three metabolites of the LOX pathway, namely, 12(S)‐hydroxyeicosatetraenoic acid (HETE), 15(S)‐HETE, and leukotriene (LT)‐B4, and three metabolites of the COX pathway, namely, prostaglandin (PG)‐D2, PGE2, and thromboxane (TX) B2, were detected in the serum of rats from the NC, RA, and MOX groups. Additionally, two metabolites of linoleic acid through the LOX/COX pathway, namely, 9(S)‐hydroxy octadecadienoic acid (HODE) and 13(S)‐HODE, were identified. Compared to NC group rats, RA group rats exhibited a significant enhancement in the serum levels of ARA and nine metabolites, thus suggesting successful establishment of the FCA‐induced RA rat model and disruption of ARA metabolism. MOX group rats showed a significant decline in the serum levels of ARA and nine metabolites as compared to RA group rats (Figure 3).

**Table 1:** The Rt, CV, and concentration results of the analytes in the QC samples.

Metabolite name	Rt (min)	CV (%)	QC‐1 (ng/mL)	QC‐2 (ng/mL)	QC‐3 (ng/mL)
Arachidonic acid	8.03	0.24	7750.384809	7760.568644	7724.774787
12(S)‐HETE	6.07	0.97	197.6041587	194.0140129	196.8942297
15(S)‐HETE	5.81	1.85	275.3904134	265.4052609	270.0152344
Leukotriene B_4_	4.27	1.14	6.606364037	6.738245004	6.60664303
Prostaglandin D_2_	2.58	0.13	12.51527184	12.52949368	12.49652143
Prostaglandin E_2_	2.57	1.36	6.492634486	6.337983805	6.485456947
Thromboxane B_2_	1.85	1.34	5.1836311	5.185946464	5.306481887
9(S)‐HODE	5.70	2.89	184.3720384	193.9358317	184.7380822
13(S)‐HODE	5.65	2.03	138.9951955	137.1466106	142.7050127

### 3.3. Inhibition of ALOX15 Promotes MOX to Improve Knee Synovial Tissue Morphology and Mitochondrial Structure in RA Model Rats

**Figure 4:**
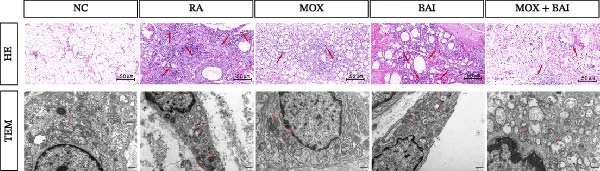
Histopathological and ultrastructural analyses of synovial tissue.

We observed an irregular cell arrangement on the surface of the synovial tissue, synovial hyperplasia and thickening, and abundant inflammatory cell infiltration in the RA group. After intervention, significant improvements were noted in synovial tissue hyperplasia and thickening and inflammatory cell infiltration in the BAI, MOX, and MOX + BAI groups. Mitochondria in synovial cells from the RA group were swollen, with unclear or fragmented cristae, reduced mitochondrial count, and vacuolar degeneration. However, following MOX intervention, mitochondrial structure showed varying degrees of recovery in the BAI, MOX, and MOX + BAI groups, with reduced mitochondrial swelling and increased mitochondrial count. These findings collectively demonstrate that inhibition of ALOX15 promotes MOX to improve knee synovial tissue morphology and mitochondrial structure in RA model rats (Figure 4).

### 3.4. Inhibition of ALOX15 Promotes MOX to Reduce Excessive Accumulation of LPOs and Suppress Ferroptosis in RA Model Rats

ALOX15, ACSL4, LPCAT3, FTH1, and GPX4 showed positive signals as yellow to tan, and their expression was detected in the cell membranes and cytoplasm of the synovial epithelium and tissue cells. IHC and WB experiments revealed markedly elevated expression levels of ALOX15, ACSL4, and LPCAT3 and remarkably reduced expression levels of GPX4 and FTH1 in the synovial tissue of RA group rats. These findings suggest the occurrence of ferroptosis and excessive accumulation of LPOs in the RA model rats. Following MOX intervention, ALOX15, ACSL4, and LPCAT3 expression levels were significantly downregulated, while FTH1 and GPX4 expression levels were substantially upregulated in the synovial tissue of rats. BAI treatment also resulted in reduced expression levels of ALOX15, ACSL4, and LPCAT3 and increased expression levels of GPX4 and FTH1 in the synovial tissue of rats. This suggests that inhibiting ALOX15 expression can, to some extent, reduce the excessive LPO accumulation and suppress ferroptosis. Notably, intervention with MOX and BAI combination exhibited more significant effects compared to MOX alone, indicating that MOX can inhibit ferroptosis by regulating ALOX15‐mediated lipid peroxidation in the RA model rats (Figure 5).

Figure 5Analysis of lipid peroxides and ferroptosis‐related proteins. (A) IHC detection of protein expression in the synovial tissue of rats from different groups (scale bar = 50 mm; *n* = 3). (B) WB detection of protein expression in the synovial tissue of rats from different groups (*n* = 3). Group differences were assessed using Kruskal–Wallis with Dunn’s post hoc.  ^∗^ *p* < 0.05,  ^∗∗^ *p* < 0.01,  ^∗∗∗^ *p* < 0.001, and  ^∗∗∗∗^ *p* < 0.0001.

**Figure:**
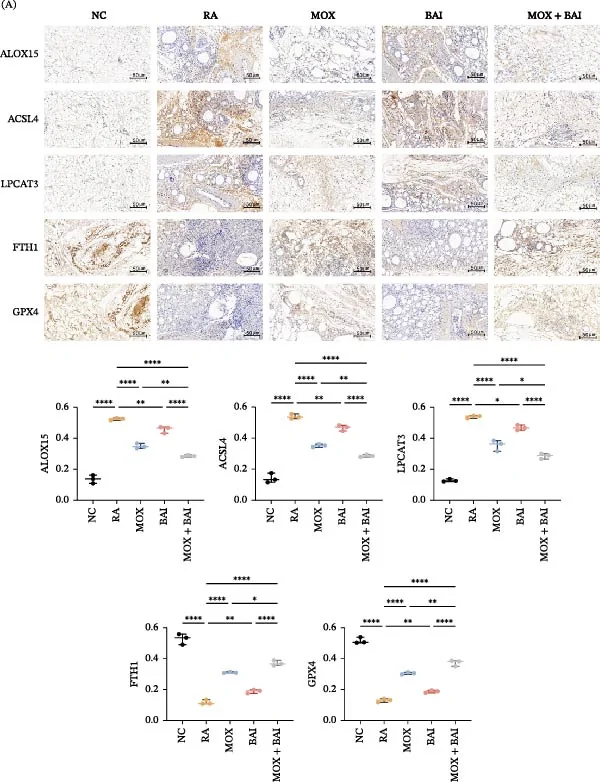

**Figure:**
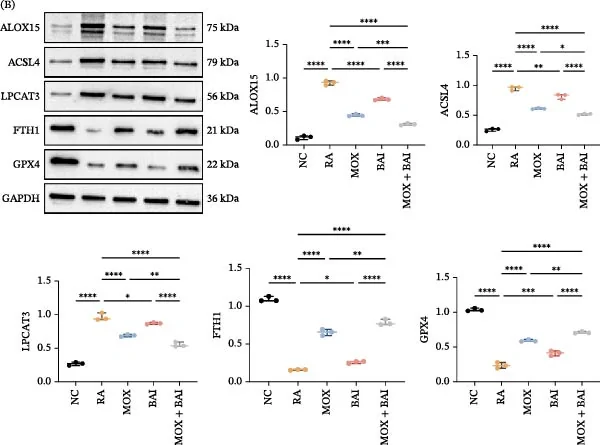

### 3.5. Inhibition of ALOX15 Promotes MOX to Alleviate Oxidative and Inflammatory Damage in RA Model Rats

**Figure 6:**
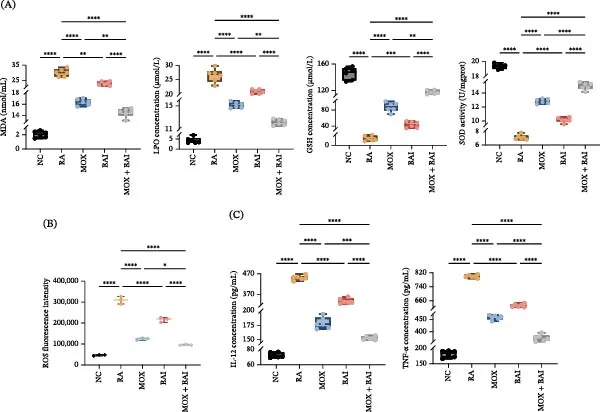
Analysis of oxidative and inflammatory damage–related indicators. (A) Biochemical detection of MDA, LPO, GSH, and SOD levels in serum (*n* = 6). (B) Flow cytometry detection of ROS levels in synovial tissue (*n* = 3). (C) ELISA detection of IL‐12 and TNF‐α levels in serum (*n* = 6). Group differences were assessed using one‐way ANOVA or Kruskal–Wallis with Dunn’s post hoc, as appropriate.  ^∗^ *p* < 0.05,  ^∗∗^ *p* < 0.01,  ^∗∗∗^ *p* < 0.001, and  ^∗∗∗∗^ *p* < 0.0001.

We noted that serum MDA, LPO, IL‐12, and TNF‐α levels were significantly elevated in RA model rats, while serum GSH and SOD levels were significantly reduced. Additionally, ROS levels in the synovial tissue were notably increased. These findings suggest a marked increase in oxidative and inflammatory damage, a decrease in antioxidant capacity, ferroptosis occurrence, and excessive LPO accumulation in RA model rats. Following MOX intervention, there was a significant decrease in MDA, LPO, ROS, TNF‐α, and IL‐12 levels and a notable elevation in GSH and SOD levels. Similarly, intervention with the ALOX15 inhibitor BAI resulted in a significant reduction in MDA, LPO, ROS, IL‐12, and TNF‐α levels and significant elevations in GSH and SOD levels. These results imply that inhibiting ALOX15 expression can alleviate the degree of oxidative and inflammatory damage, restore antioxidant capacity, inhibit ferroptosis, and reduce LPO accumulation to some extent (Figure 6).

## 4. Discussion

RA is an autoimmune disease that primarily manifests as pannus formation, synovial intimal hyperplasia, cartilage destruction, and systemic complications [17]. Nonsteroidal anti‐inflammatory drugs, disease‐modifying antirheumatic drugs, glucocorticoids, and biologics are frequently used to treat RA in clinical settings. While these treatments offer some anti‐inflammatory and analgesic effects, these effects are associated with numerous adverse reactions and impose a significant economic burden [18, 19]. Therefore, safe, effective, affordable, and minimally side effect–prone complementary and alternative medical approaches should be urgently developed for treating RA.

In TCM, RA is categorized as “Bi syndrome,” with complex pathogenic evolution and diverse clinical manifestations [20, 21]. According to the “Yellow Emperor’s Inner Canon,” the combination of wind, cold, and dampness leads to Bi syndrome [22]. The main pathogenic mechanism of RA in TCM is understood as “insufficiency of Zheng Qi, invasion of wind, cold, and dampness, and stagnation of Qi and blood.” The main functions of the Zusanli (ST36) acupoint include invigorating the stomach and spleen, regulating blood and Qi, promoting meridian circulation, and strengthening the body’s resistance. Chen et al. [23] found that acupuncture at Zusanli (ST36) can modulate pentose phosphate and ARA metabolic pathways and significantly improve inflammatory responses in RA model mice. Shenshu (BL23) acupoint is primarily used to benefit the kidneys and regulate water metabolism. Haiyan discovered that acupuncture at Shenshu can enhance immune suppression by decreasing the serum TNF‐α level [24]. MOX is a crucial element of TCM, with effects such as dispersing cold and eliminating dampness, dispersing wind and dredging meridians, and promoting Qi and blood circulation [25]. Previous studies suggest that MOX at Zusanli (ST36) and Shenshu (BL23) significantly alleviates synovial inflammatory damage in arthritis model rats by suppressing inflammatory cytokine and related signaling pathway molecule release [26–28].

ARA serves as a precursor for various bioactive substances and has a critical function in the synthesis of proinflammatory mediators [29–32]. ARA metabolism occurs mainly through three pathways: COX, LOX, and CYP450, generating different metabolites. These metabolites participate in various inflammatory states related to RA and contribute to progressive cartilage and bone destruction [33, 34]. To elucidate the changes in ARA levels during the development of RA and to understand the primary pathways through which MOX improves RA by regulating ARA metabolism, this study conducted a targeted metabolomics analysis of ARA in the serum of rats with RA and those treated with MOX. ARA metabolism was disordered in RA model rats. After MOX intervention, a decrease was noted in serum levels of ARA and its metabolites, including 12(S)‐HETE, 15(S)‐HETE, LTB4, PGD2, PGE2, TXB2, 13(S)‐HODE, and 9(S)‐HODE. Among these, the change in 15(S)‐HETE was the most significant. The generation process of 15(S)‐HETE involved ALOX15‐catalyzed oxidation of the 15th carbon of ARA, resulting in the intermediate product 15‐hydroperoxyeicosatetraenoic acid (15‐HPETE), which promoted ferroptosis [35]. Subsequently, under the action of peroxidases such as GPX4, 15‐HPETE was reduced to 15(S)‐HETE [36]. Therefore, we intervened in RA model rats using the ALOX15 inhibitor BAI and conducted subsequent studies.

**Figure 7:**
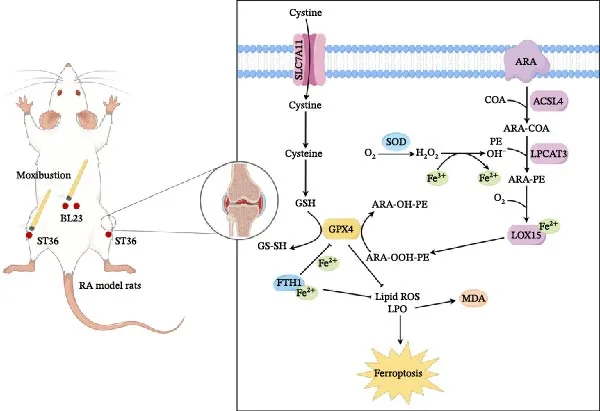
Mechanism diagram of the regulation of ALOX15‐mediated LPO by moxibustion to inhibit ferroptosis and mitigate synovial inflammatory injury in RA model rats.

Ferroptosis relies on the accumulation of LPOs, which primarily originate from PUFAs, including ARA [37]. Inflammatory mediators produced by lipid peroxidation and ARA metabolism are present in ferroptosis tissues [38]. Ferroptosis can accelerate ARA metabolism and promote the secretion of inflammatory signaling molecules [39]. The metabolism of both amino acid and iron in the ferroptosis pathway ultimately causes changes in lipid metabolism, suggesting that inhibiting ferroptosis can be effectively achieved by intervening in lipid peroxidation‐related targets. Lipid peroxidation has a key function in RA pathogenesis and progression [40]. ACSL4 and LPCAT3 are key enzymes involved in regulating the metabolic conversion of PUFAs, such as ARA. Under the catalysis of ALOX15, these substrates are oxidized to generate lipid hydroperoxides, which occupy a central position in the lipid metabolism regulatory network of ferroptosis [41]. By combining targeted metabolomics results, this study further explores some of the mechanisms of MOX treatment for RA from the perspective of ferroptosis suppression through ALOX15‐mediated lipid peroxidation inhibition. Excessive accumulation of Fe^2+^ can lead to lipid peroxidation damage. FTH1, an iron storage protein possessing ferroxidase activity, converts Fe^2+^ to Fe^3+^ and has an essential function in sustaining iron metabolism homeostasis [42]. GPX4 directly minimizes the production of peroxidized phospholipids on the cell membrane and scavenges excessive oxides in the body [43]. MDA is the final product generated through the enzymatic or nonenzymatic breakdown of ARA and larger PUFAs [44]. Dysregulation of lipid synthesis and metabolism results in the accumulation of PUFAs, which consumes a significant amount of intracellular GSH, leading to the generation of more peroxidized lipids and increasing cell sensitivity to undergo ferroptosis. Following GPX4 or GSH depletion, ROS accumulation directly leads to lipid peroxidation induction and ferroptosis activation in cells [45]. These changes in oxidative and inflammatory biomarkers align with the observed suppression of ALOX15‐mediated lipid peroxidation and ferroptosis, providing a coherent evidence chain (Figure 7).

## 5. Conclusions

In summary, MOX at Zusanli (ST36) and Shenshu (BL23) acupoints mitigated inflammation and damage of the synovial tissue in RA model rats. The underlying mechanism might be closely associated with ALOX15‐mediated lipid peroxidation regulation, mitigation of oxidative damage, reduction of LPO accumulation, subsequent inhibition of ferroptosis, attenuation of inflammatory responses, and slowdown of joint cartilage destruction. Lipid peroxidation and ferroptosis occurrence involve the expression and regulation of multiple genes and pathways, and further investigation is required to determine whether MOX inhibits ferroptosis through other regulatory factors.

## Author Contributions

**Tiancheng Wang**, **Chuanyue Peng**, and **Qiannan Liu**: data curation, formal analysis, methodology, visualization, writing – original draft. **Zanchen Zhou** and **Dong Gao**: investigation, supervision, validation, data curation. **Yifan Li** and **Meilin Zhang**: investigation, supervision, data curation. **Feng Hao** and **Chuanyu Peng**: conceptualization, funding acquisition, methodology, project administration, software, supervision, validation, writing – review and editing.

## Funding

This work was supported by grants from the National Natural Science Foundation of China (Grants 82205289 and 82274645) and the Natural Science Research Project of Anhui Educational Committee (Grant 2025AHGXZK31403).

## Disclosure

All authors contributed to the article and approved the submitted version.

## Ethics Statement

The Animal Ethics Committee of Anhui University of Chinese Medicine approved this study (Approval Number AHUCM‐rats‐2024013).

## Conflicts of Interest

The authors declare no conflicts of interest.

## Supporting Information

Additional supporting information can be found online in the Supporting Information section.

## Supporting information


**Supporting Information** Table S1: Ion pair information for target substances.

## Data Availability

The data that support the findings of this study are available from the corresponding author upon reasonable request.
