# Supplementary material for: Moxibustion Modulates ALOX15‐Mediated Lipid Peroxidation to Inhibit Ferroptosis in Synovial Inflammatory Injury of Rheumatoid Arthritis
Source: Mediators Inflamm. 2026 Jul 2;2026:4516938. doi: 10.1155/mi/4516938 (PMC13329117; doi:10.1155/mi/4516938)
Supplement: Supplementary file 1 — Supporting Information Table S1: Ion pair information for target substances. [file MI-2026-4516938-s001.docx]

**Table S1**. Ion Pair Information for Target Substances.

| **Metabolite Name** | **Mass Info** | **HMDB ID** | **CAS** |
| --- | --- | --- | --- |
| Arachidonic acid | 303.2 / 259.2 | HMDB0001043 | 506-32-1 |
| Docosahexaenoic acid | 327.1 / 229.2 | HMDB0002183 | 6217-54-5 |
| 12(S)- HETE | 319.1 / 179.0 | HMDB0006111 | 54397-83-0 |
| 15(S)-HETE | 319.1 / 219.0 | HMDB0003876 | 54845-95-3 |
| Leukotriene B_4_ | 335.2 / 195.0 | HMDB0001085 | 71160-24-2 |
| Leukotriene D_4_ | 495.3 / 177.2 | HMDB0003080 | 73836-78-9 |
| Prostaglandin D_2_ | 351.2 / 271.3 | HMDB0001403 | 41598-07-6 |
| Prostaglandin E_2_ | 351.2 / 271.3 | HMDB0001220 | 363-24-6 |
| Prostaglandin F_2α_ | 353.1 / 309.3 | HMDB0001139 | 551-11-1 |
| Thromboxane B_2_ | 369.2 / 169.0 | HMDB0003252 | 54397-85-2 |
| 6-keto-Prostaglandin F_1α_ | 369.3 / 163.0 | HMDB0002886 | 58962-34-8 |
| 8-iso-Prostaglandin F_2α_ | 353.1 / 309.3 | HMDB0005083 | 27415-26-5 |
| 9(S)-HODE | 295.1 / 171.1 | HMDB0004670 | 73543-67-6 |
| 13(S)-HODE | 295.0 / 195.0 | HMDB0004667 | 29623-28-7 |
